# Generation and characterization of transgenic mice expressing mitochondrial targeted red fluorescent protein selectively in neurons: modeling mitochondriopathy in excitotoxicity and amyotrophic lateral sclerosis

**DOI:** 10.1186/1750-1326-6-75

**Published:** 2011-11-02

**Authors:** Yi Wang, Yan Pan, Ann Price, Lee J Martin

**Affiliations:** 1 Department of Pathology, Division of Neuropathology, Johns Hopkins University School of Medicine, Baltimore, Maryland, MD 21205, USA

**Keywords:** Alzheimer's disease, amyotrophic lateral sclerosis, CA1 neuron, excitotoxicity, motor neuron, Parkinson's disease

## Abstract

**Background:**

Mitochondria have roles or appear to have roles in the pathogenesis of several chronic age-related and acute neurological disorders, including Charcot-Marie-Tooth disease, amyotrophic lateral sclerosis, Parkinson's disease, and cerebral ischemia, and could be critical targets for development of rational mechanism-based, disease-modifying therapeutics for treating these disorders effectively. A deeper understanding of neural tissue mitochondria pathobiologies as definitive mediators of neural injury, disease, and cell death merits further study, and the development of additional tools to study neural mitochondria will help achieve this unmet need.

**Results:**

We created transgenic mice that express the coral (*Discosoma sp*.) red fluorescent protein DsRed2 specifically in mitochondria of neurons using a construct engineered with a Thy1 promoter, specific for neuron expression, to drive expression of a fusion protein of DsRed2 with a mitochondrial targeting sequence. The biochemical and histological characterization of these mice shows the expression of mitochondrial-targeted DsRed2 to be specific for mitochondria and concentrated in distinct CNS regions, including cerebral cortex, hippocampus, thalamus, brainstem, and spinal cord. Red fluorescent mitochondria were visualized in cerebral cortical and hippocampal pyramidal neurons, ventrobasal thalamic neurons, subthalamic neurons, and spinal motor neurons. For the purpose of proof of principle application, these mice were used in excitotoxicity paradigms and double transgenic mice were generated by crossing Thy1-mitoDsRed2 mice with transgenic mice expressing enhanced-GFP (eGFP) under the control of the *Hlxb9 *promoter that drives eGFP expression specifically in motor neurons and by crossing Thy1-mitoDsRed2 mice to amyotrophic lateral sclerosis (ALS) mice expressing human mutant superoxide dismutase-1.

**Conclusions:**

These novel transgenic mice will be a useful tool for better understanding the biology of mitochondria in mouse and cellular models of human neurological disorders as exemplified by the mitochondrial swelling and fission seen in excitotoxicity and mouse ALS.

## Background

Mitochondria have been implicated in the pathobiology of several neurological disorders, including Charcot-Marie-Tooth disease, chronic progressive external opthalmoplegia, mitochondrial encephalomyopathy lactic acidosis and stroke-like episodes syndrome, and less directly in stroke, amyotrophic lateral sclerosis (ALS), Parkinson's disease, and Alzheimer's [1-4]. A variety of pathogenic mechanisms could be directly or indirectly linked to perturbations in mitochondrial physiology and signaling [3,4], notably trafficking, fission/fusion, oxidative stress and reactive oxygen species (ROS) damage to macromolecules, glutamate receptor excitotoxicity and intracellular Ca^2+ ^deregulation, protein aggregation, and permeability transition pore activation [5,6]. Mitochondria are producers of toxic ROS that can damage cellular constituents, including DNA, RNA, protein, and lipids, and initiate many forms of cell death in mammalian cells [2,6,7]. Human mutant proteins linked to Parkinson's disease, ALS, and Alzheimer's disease can associate with mitochondria in mouse and cellular models and can cause mitochondrial dysfunction [3,6]. Mitochondria are sources of several apoptogenic proteins that upon release execute the apoptotic process [6-8]. Release of apoptogenic proteins from mitochondria can occur through mechanisms involving formation of membrane channels comprised of Bax [9], Bax and the adenine nucleotide translocator [10], the voltage dependent anion channel (VDAC) [11], and the mitochondrial permeability transition pore (mPTP) [7]. Mitochondria mediate the apoptotic process in adult brain neurons by mPTP-triggered ROS and nitric oxide production after their accumulation and priming instigated by Zn^2+ ^and Ca^2+ ^accumulation [12]. Mitochondrial targeted drugs such as TRO19662 (olesoxime) and Bcl-X_L_:BH4 peptides can block apoptosis of neurons within the adult mouse CNS [12]. Thus, mitochondria are validated important targets for the design of drugs and small molecules as neuroprotectants with potential *in vivo *CNS efficacy in the treatment of several neurological disorders [6,12].

The ability to genetically express jellyfish and coral fluorescent proteins in mammalian cells has revolutionized experimental approaches [13]. Under the control of specific gene promoters, fluorescence proteins can serve as reporters for tissues, cells, and organelles, as well as toxicity biosensors [14-16]. Many transgenic (tg) mouse lines have been engineered to express green fluorescent protein (GFP), yellow fluorescent protein (YFP), red fluorescent protein, or cyan fluorescent protein in tissues and cells. For example, motor neuron and neuromuscular junction (NMJ) biology can be studied using tg mice expressing enhanced-GFP (eGFP) under the control of the *Hlxb9 *promoter that drives eGFP expression specifically in motor neurons and their entire cell body and peripheral axon to the NMJ [17]. The NMJ can also be evaluated in tg mice expressing YFP driven by the neuron-specific promoter thymus cell antigen 1(*Thy1) *[18,19]. In these mice, neurons are filled with YFP to reveal a Golgi stain-like resolution [18]. Interneurons in brain and spinal cord are difficult to identify with certainty in tissue sections using antibodies and in living slices and dissociated cell cultures, but in tg mice expressing eGFP driven by the *glutamic acid decarboxylase *gene promoter [20] or the *glycine transporter-2 *gene promoter [21], GABAergic and glycinergic interneurons, respectively, can be visualized in exquisite detail. Here, we describe the generation and characterization of new tg mice that express the marine coral (*Discosoma sp*.) red fluorescent protein DsRed2 specifically in mitochondria of neurons.

## Results

### Engineering of the Thy1-mitoDsRed gene construct

**Figure 1:**
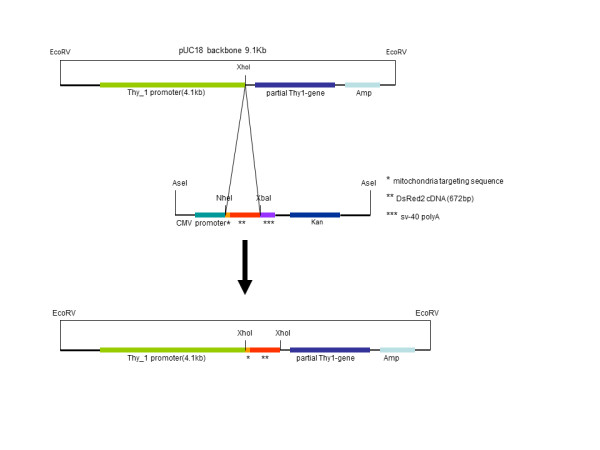
**Design of Thy1-mitoDsRed construct used to generate tg mice**. A tg construct containing the entire open reading frame of the *DsRed2 *gene (red) fused in frame with the mitochondrial targeting sequence of the human *cytochrome c oxidase subunit VIII *gene (orange) was cloned into the Thy1.2 expression cassette at the XhoI site by T4 ligation.

The design of the Thy1-mitoDsRed2 expression construct is shown in Figure 1. We cloned the entire fragment of mitoDsRed2 gene into the XhoI site of the Thy1 promoter by T4 ligation (see Methods). Correct clones were identified by restriction enzyme digestion and direct sequencing (data not shown). All sequences were confirmed by sequencing in both forward and reverse directions. In addition, cultured cells were transfected with the construct to confirm that the codons are in-frame as determined by protein expression.

### Visualization of mitochondria in living cultured neurons using mitochondrial-targeted DsRed2

**Figure 2:**
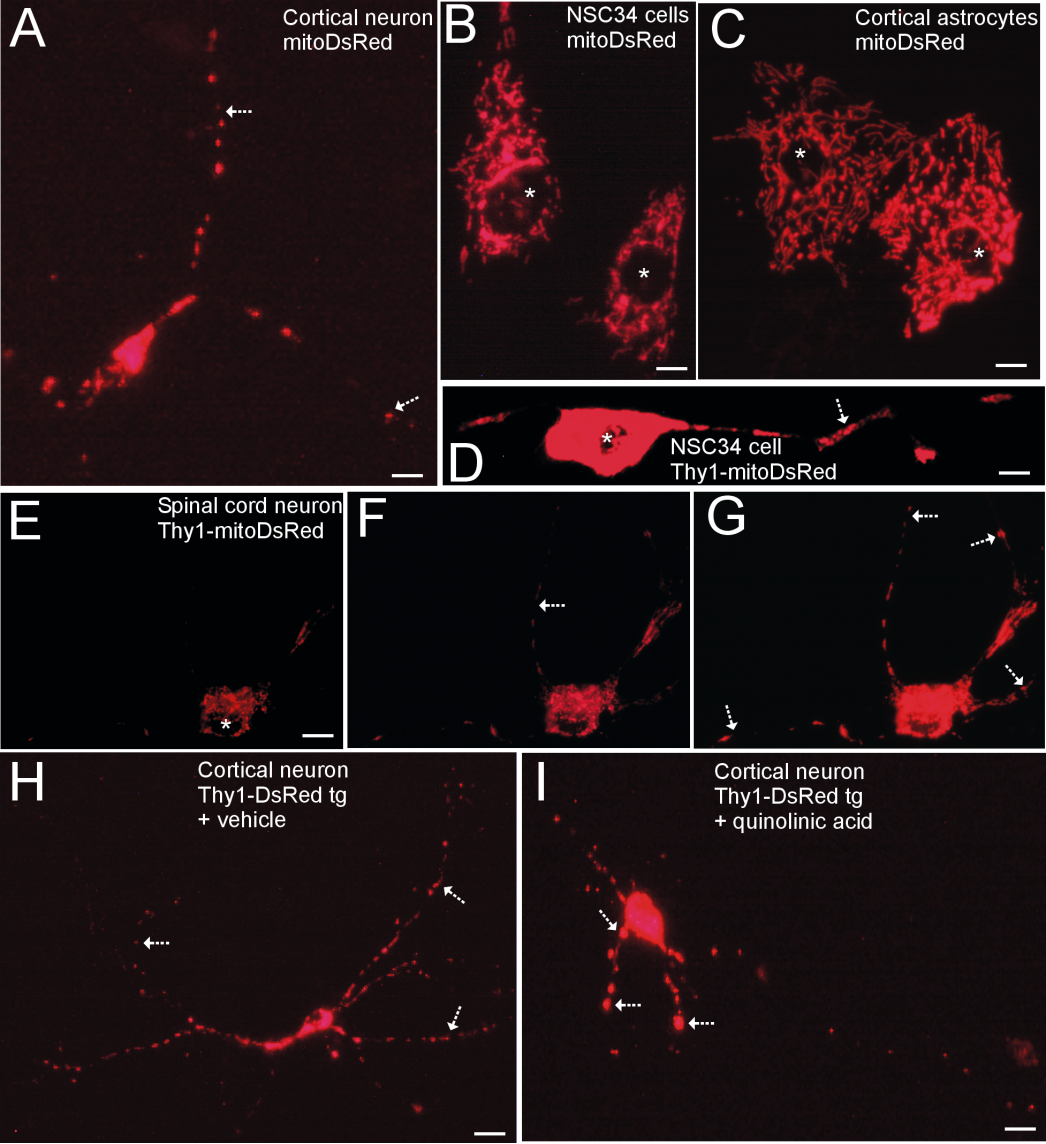
**Visualization of mitochondria in living cultured neurons using mitochondrial-targeted DsRed2**. **A**. Embryonic mouse primary cerebral cortical neuron transfected with mitoDsRed plasmid. The cell body (lower left) containing numerous mitochondria is overexposed to show individual mitochondria in fine distal processes (arrows). Scale bar = 7 μm. **B**. NSC34 motor neuron-like cells transfected with mitoDsRed plasmid showing vast numbers of mitochondria forming a network in the cytoplasm surrounding the nucleus (asterisk). Scale bar = 6 μm. **C**. Mouse cerebral cortical astrocytes expressing mitochondrial-targeted DsRed show mitochondria as long vermiform, ellipsoid, or round organelles. Asterisks identify nucleus. Scale bar = 6 μm. **D**. NSC34-motor neuron transfected with Thy1-mitoDsRed plasmid. Cell body fluorescence is bright due to numerous mitochondria and overexposure, while individual mitochondria in a long dendrite (arrow) can be discerned. Scale bar = 6 μm. **E-G**. Embryonic mouse primary spinal cord neuron transfected with Thy1-mitoDsRed plasmid and shown at different exposures to reveal red fluorescent mitochondria in the cell body (E, asterisk marks the nucleus) and distal processes (F and G, arrows). Scale bar = 6 μm. **H and I**. NMDA receptor activation by quinolinic acid causes mitochondrial pathology as seen directly in living cultured primary cortical neurons by Thy1-mitoDsRed. Mouse cortical neurons treated with PBS (vehicle) show numerous individual mitochondria distributed throughout the dendritic arborization (H, arrows). Neurons treated with 100 μM quinolinic acid undergo mitochondrial swelling (arrows) and dendrite retraction by 4 hours (I). Scale bars = 6 μm. Images are representative of at least 3 different cell culture experiments.

To establish a pattern for recognition of DsRed-labeled mitochondria in living cells, we used the unmodified mitoDsRed2 plasmid (Clontech) to transfect mouse primary cortical neurons and motor neuron-like cells differentiated from the NSC34 cell line. Transfected cortical neurons (Figure 2A) and NSC34 cells (Figure 2B) showed bright red fluorescence as discrete round-, oval-, and rod-like foci consistent with expectations for mitochondria and with previous reports [22-24]. DsRed-labeled mitochondria could be tracked far distances within the processes of cortical neurons (Figure 2A, arrows). Large differentiated motor neuron-like cells in NSC34 cell cultures showed numerous perikaryal mitochondria (Figure 2B). Astrocytes in mixed-cell cortical cultures transfected with mitoDsRed2 also displayed vast numbers of mitochondria (Figure 2C).

Cell cultures transfected with our Thy1-mitoDsRed plasmid showed fluorescent labeling in a pattern consistent with mitochondria, confirming that the design of the engineered plasmid was correct by direct demonstration of expressed DsRed protein. Motor neurons differentiated from NSC34 cells showed high expression of Thy1-mitoDsRed in the cell bodies and far into distal processes (Figure 2D). Similarly, living primary spinal cord neurons transfected with Thy1-mitoDsRed showed fluorescent mitochondria in the cell body (Figure 2E) and, with longer exposures, in numerous fine distal processes (Figure 2F, G). Transfected astrocytes were not seen in cortical neuron and spinal cord neuron cultures transfected with Thy1-mitoDsRed. To model an insult that would be expected to produce mitochondrial pathology in neurons, an excitotoxic challenge was used [24]. Cortical neuron cultures transfected with Thy1-mitoDsRed and treated with the potent N-methyl-D-aspartate (NMDA) receptor agonist quinolinic acid showed swelling of mitochondria and dendritic attrition (Figure 2I, arrows) in comparison with transfected neurons treated with vehicle (Figure 2H).

### Thy1-mitoDsRed2 tg mouse generation and characterization of tissue-specific expression of DsRed2 in tg mice using immunoblotting and RT-PCR

**Figure 3:**
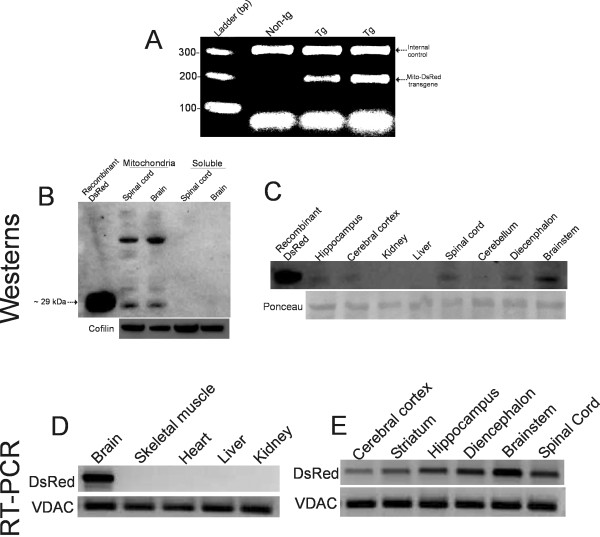
**Thy1-mitoDsRed2 tg mouse generation and characterization**. **A**. Genomic identification of tg mice. Thy1-mitoDsRed tg mice showed a PCR-amplified 208 bp product that was undetected in non-tg mice. **B**. Western blot analysis of subcellular fractions of Thy1-mitoDsRed tg mouse brain and spinal cord demonstrating the presence of DsRed protein in mitochondrial-enriched fractions but not in soluble protein fractions. Purified recombinant DsRed was loaded as a positive control. Blots were reprobed with antibody to cofilin to show protein loading. Results were replicated using several tg mice (n = 8). **C**. Western blot analysis showing the tissue distributions and brain regional distributions of DsRed protein expression in Thy1-mitoDsRed tg mice. Crude extracts were used. DsRed protein was detected at varying levels of expression in most CNS regions but not in liver or kidney (4 of 5 lines). Recombinant DsRed was loaded as a positive control. The nitrocellulose membrane was stained with Ponceau S to show protein loading. Results were replicated using several tg mice (n = 8). **D**. RT-PCR analysis showing the tissue distributions of DsRed mRNA expression in Thy1-mitoDsRed tg mice. DsRed mRNA was detected in brain but not in skeletal muscle, heart, liver, or kidney. VDAC1 mRNA was used as an internal control. Results were replicated using several tg mice (n = 5). **E**. RT-PCR analysis showing brain regional distributions of DsRed mRNA expression in Thy1-mitoDsRed tg mice. DsRed protein was detected at varying levels of expression in several different CNS regions. VDAC1 mRNA was an internal control. Results were replicated using several tg mice (n = 5).

We had 5 tg founder mice for Thy1-mitoDsRed 2 identified by PCR amplification of the DsRed transgene (Figure 3A) which were used to develop a mouse colony. Western blotting was used to establish the mitochondrial expression of DsRed in brain and spinal cord tissue fractions. In Thy1-mitoDsRed2 tg mice, DsRed protein was concentrated in mitochondrial-enriched fractions of whole brain and spinal cord but was undetectable in soluble protein fractions of these tissues (Figure 3B). In brain and spinal cord mitochondria, monomeric DsRed migrated at ~29 kDa, matching the migration of recombinant DsRed (Figure 3B). The DsRed monoclonal antibody also detected higher molecular proteins in the mitochondrial fractions that were not seen in soluble fractions (Figure 3B), but the identity of these reactive proteins is unclear. Western blotting for DsRed in crude tissue extracts of CNS and body organs of Thy1-mitoDsRed tg mice, compared against recombinant DsRed, confirmed the nervous tissue-specific expression of DsRed. In four of five tg mouse lines, DsRed was detected in cerebral cortex, hippocampus, diencephalon, brainstem, cerebellum, and spinal cord, but not in kidney and liver (Figure 3C). Other organs evaluated that were negative for DsRed were heart and skeletal muscle (data not shown). One of five tg mouse lines showed low expression of DsRed protein in kidney (data not shown) in addition to CNS expression. RT-PCR was used to show mRNA expression specifically in brain tissue. DsRed mRNA was detected robustly in brain but not in skeletal muscle, heart, liver, and kidney in 4 of 5 lines (Figure 3D). DsRed mRNA expression was further shown in specific regional microdissections of cerebral cortex, hippocampus, striatum, diencephalon, brainstem, and spinal cord (Figure 3E). The brainstem showed the highest level of DsRed gene expression of these regions (Figure 3E).

### Neuronal mitochondria visualization in Thy1-mitoDsRed2 tg mouse brain and spinal cord

**Figure 4:**
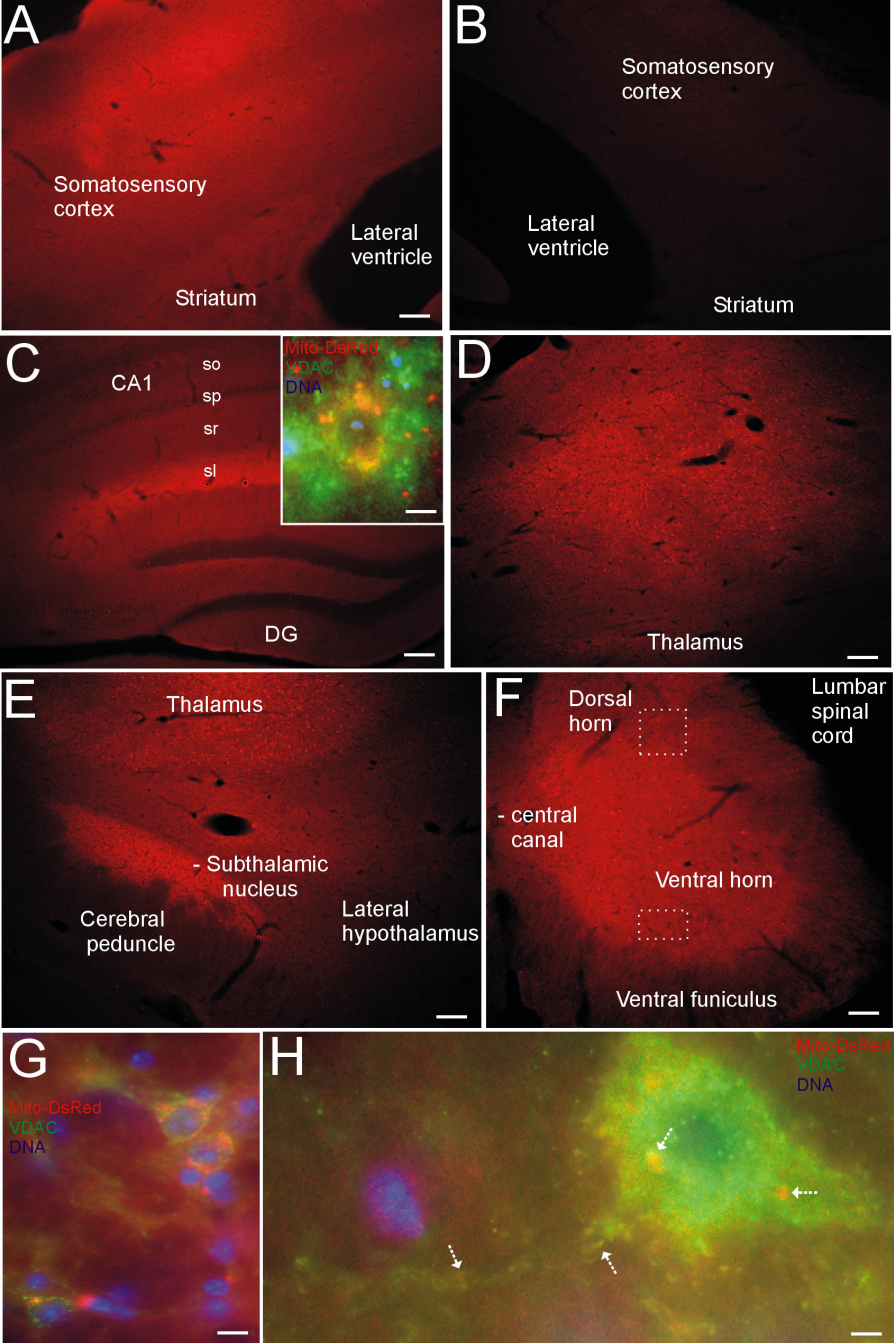
**Mitochondrial visualization in Thy1-mitoDsRed2 tg mouse CNS**. **A**. Tg mice showed DsRed fluorescence in somatosensory cortex with varying intensities in different cortical layers. Scale bar = 70 μm (same for B). **B**. Non-tg mice have no red fluorescence in somatosensory cortex. **C**. Tg mice showed DsRed fluorescence in hippocampus. All layers of CA1 were fluorescent, including stratum oriens (so), stratum pyramidale (sp), stratum radiatum (sr), and stratum lacunosum-moleculare (sl), with the sl showing the highest intensity; the dentate gyrus (DG) showed lower DsRed fluorescence compared to CA1. The granule cell layer of DG was nearly negative. Inset shows colocalization (orange-yellow) of DsRed fluorescence with VDAC (green) in a CA1 pyramidal neuron cell body. Nuclei are blue. Scale bars = 47 μm (inset, 4 μm). **D**. Tg mice showed DsRed fluorescence in ventrobasal complex of thalamus. Scale bar = 70 μm. **E**. Tg mice showed DsRed fluorescence in subthalamic nucleus, while nearby cerebral peduncle and lateral hypothalamus showed very low fluorescence. Scale bar = 70 μm. **F**. Spinal cord gray matter showed DsRed fluorescence in tg mice. White hatched boxes in dorsal horn and ventral delineate regions shown at higher magnification in G and H. Scale bar = 100 μm. **G**. Colocalization (orange-yellow) of DsRed fluorescence with VDAC (green) in dorsal horn neurons. Cell nuclei are blue. Scale bar = 5 μm. **H**. Colocalization (orange-yellow, arrows) of DsRed fluorescence with VDAC (green) in motor neuron. Scale bar = 3 μm. Images are representative of numerous tg (n = 20) or control (n = 10) mice.

Thick sections (40 μm) from tg and non-tg littermate mouse brain and spinal cord were examined for DsRed fluorescence. Generally, fluorescence for DsRed was observed highest in cerebral cortex, hippocampus, striatum, diencephalon, brainstem, and spinal cord but was very low in regions such as the hypothalamus (Figure 4). Younger mice (< 6 months of age) generally showed stronger DsRed fluorescence than older mice (> 12 months of age). DsRed fluorescence was much more obvious in gray matter than in white matter. In somatosensory cortex, mid-layers showed extremely bright DsRed fluorescence (Figure 4A). In the homotypic cortex of non-tg littermate mice no red fluorescence was seen (Figure 4B), serving as a negative control. In hippocampus, intense DsRed fluorescence was present in the stratum lacunosum-moleculare of CA1 (Figure 4C), a site of synaptic termination of entorhinal perforant path fibers and Schaffer collaterals onto distal dendrites of CA1 pyramidal neurons [25], but, in comparison, the adjacent dentate gyrus had low DsRed fluorescence (Figure 4C). Under high magnification, individual CA1 pyramidal neuron cell bodies contained numerous DsRed-tagged mitochondria that co-labeled with VDAC immunoreactivity (Figure 4C, inset) or SOD2 immunoreactivity (data not shown). The thalamus, particularly the ventrobasal complex, contained numerous neurons expressing DsRed (Figure 4D, E) and the nearby subthalamic nucleus was also highly enriched in DsRed (Figure 4E). The spinal cord gray matter neuropil was brightly fluorescent for DsRed (Figure 4F). Subsets of individual neurons deep within the dorsal horn possessed abundant DsRed-labeled mitochondria, confirmed as mitochondria by VDAC immunoreactivity (Figure 4G). Ventral horn motor neurons also contained numerous perikaryal and dendritic mitochondria that co-labeled with DsRed and VDAC immunoreactivity. Interestingly, in hippocampal and spinal cord neurons, not all DsRed-tagged mitochondria were co-labeled with mitochondrial marker immunoreactivity and vice versa (Figure 4C inset, G, H).

**Figure 5:**
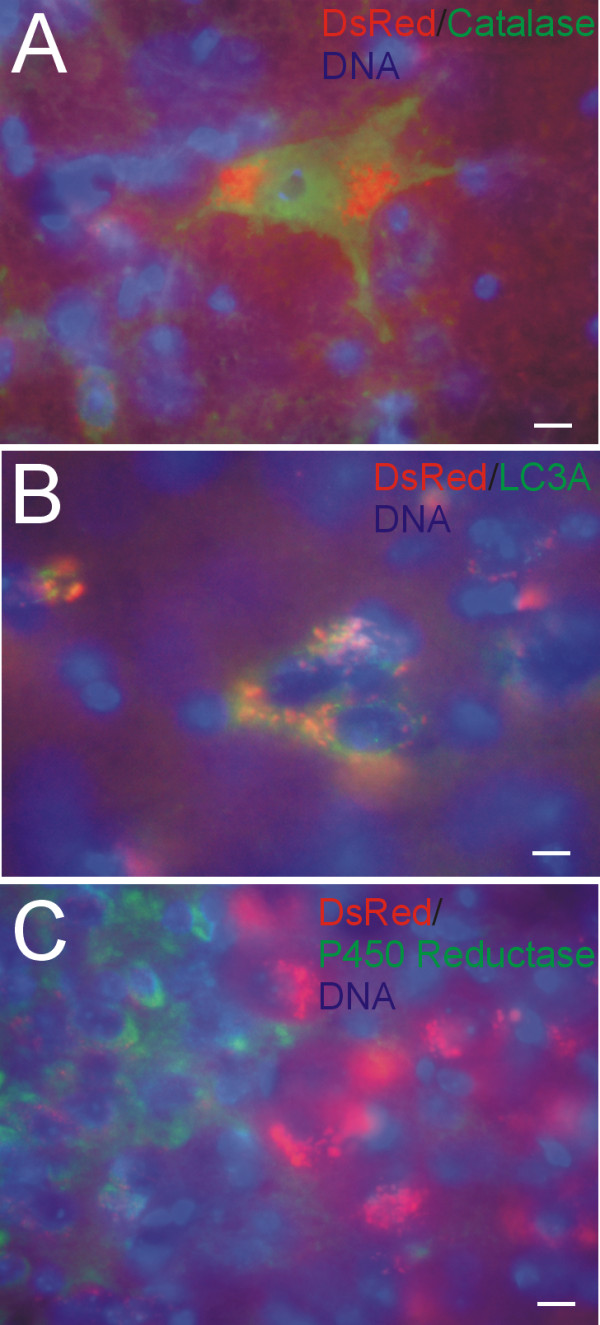
**Further characterization of the intracellular localization of DsRed in neurons Thy1-mitoDsRed2 tg mice**. Immunofluorescent localization of catalase (A), LC3A (B) and cytochrome P450 reductase (C), as markers for peroxisomes/nucleus, autophagic vesicles and endoplasmic reticulum (ER), respectively, in brainstem sections shows mitochondrial-specific labeling with DsRed. Cell nuclei (blue) were visualized with the DNA stain Hoechst-33342 dye. **A**. Large brainstem neurons showing intracellular segregation of catalase immunoreactivity (green) and DsRed. Scale bar = 7 μm. **B**. Brainstem neurons showing partial overlap of cytoplasmic DsRed and LC3A (green) seen as yellow. Individual red and green signals are also seen. Scale bar = 5 μm. **C**. Brainstem locus ceruleus neurons (red, right) peri-cerulean neurons showing distinct DsRed and ER (green) intracellular localizations. Scale bar = 5 μm. Images are representative of several different tg mice (n = 8).

Immunohistochemistry was used to characterize further the intracellular localization of DsRed in Thy1-mitoDsRed tg mouse brain sections by immunofluorescence (Figure 5). DsRed fluorescence in neurons was segregated from the peroxisome compartment and nucleus as demonstrated by localizations distinct from catalase (Figure 5A). Interestingly, DsRed did show some colocalization with the autophagy marker LC3A (Figure 5B), possibly reflecting mitophagy. In contrast, intracellular DsRed was completely distinct from the smooth endoplasmic reticulum (ER) compartment, as indicated by the lack of overlap with cytochrome P450 reductase (Figure 5C).

### Thy1-mitoDsRed2 tg mice display no overt neurological abnormalities or neuropathology

Thy1-mitoDsRed tg mice are healthy and fertile. They have a normal lifespan of > 24 months. They show no overt evidence of seizure abnormalities or movement disorders. Brain and spinal cord sections examined using Nissl and silver staining, as well as immunofluorescent staining for cleaved caspase-3, showed no evidence for neurodegeneration (data not shown).

### Applications of Thy1-mitoDsRed2 tg mice in studies of motor neurons

**Figure 6:**
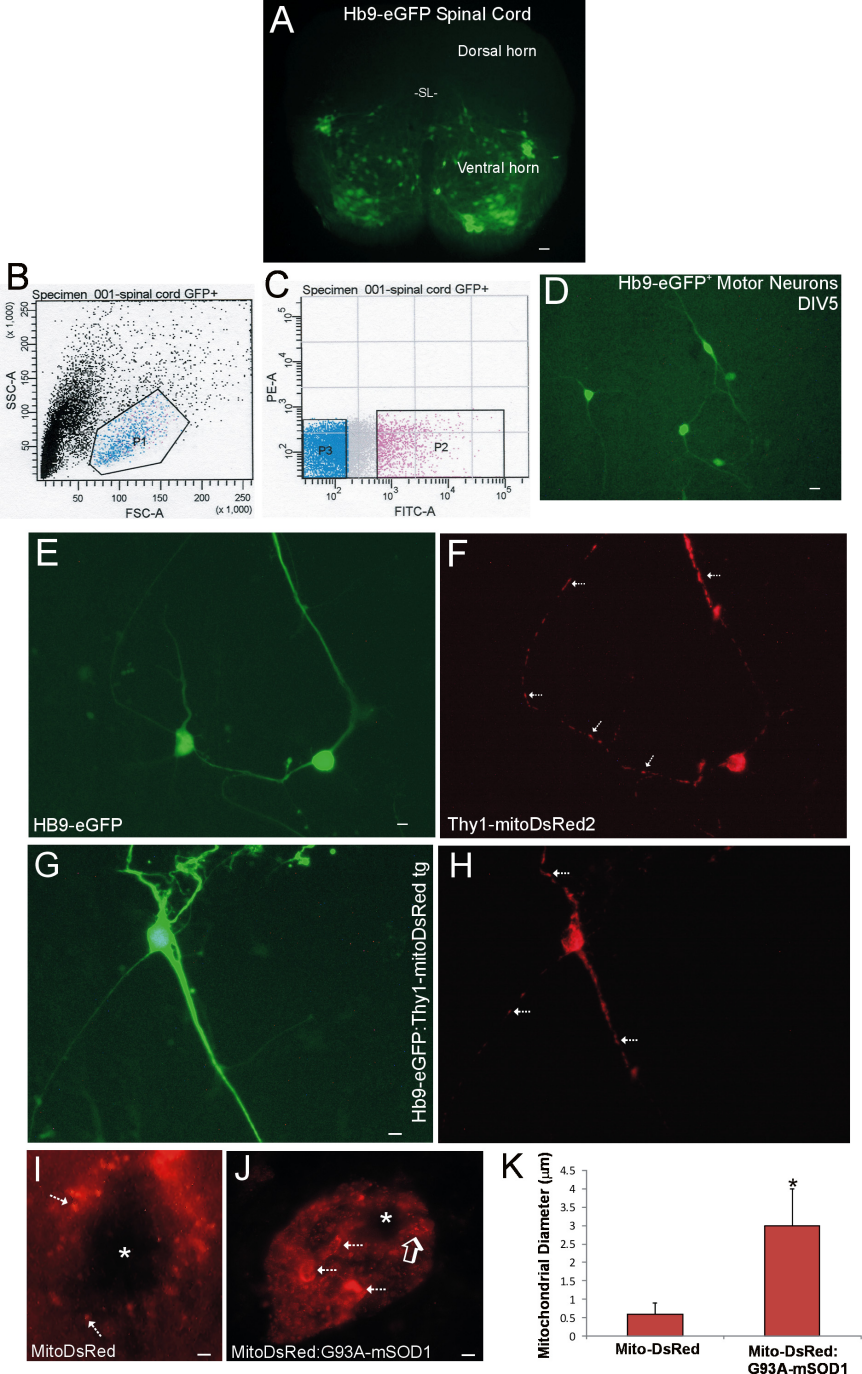
**Thy1-mitoDsRed2 tg mice in studies of motor neurons**. **A**. E13 tg Hb9-eGFP mouse embryo section showing motor neurons below the sulcus limitans (SL). Scale bar = 40 μm. **B**. Spinal cords of E13 Hb9-eGFP tg mouse embryos were subjected to flow cytometry and sorted by side scatter (SSC) and forward scatter (FSC) into small cells/debris and large cells (P1, blue and red dots). **C**. P1 (large) cells were subjected to FACS and sorted by viability based on phycoerythrin annexin (PE-A) negativity and eGFP (FITC)-positivity resulting in P2 cells. **D**. Cultured P2 cells were eGFP-positive motor neurons. Scale bar = 15 μm. **E, F**. Mitochondria (F, arrows) in living Hb9-eGFP motor neurons visualized by transfection with Thy1-mitoDsRed2 construct. Scale bar = 7 μm. **G, H**. Double tg mice expressing Hb9-eGFP and Thy1-mitoDsRed2 were created to image mitochondria (arrows) directly in living motor neurons. Scale bar = 7 μm. **I, J**. Mitochondrial visualization (hatched arrows) in situ in spinal cord motor neurons of Thy1-mitoDsRed tg mice (I) and tg ALS mice expressing human mutant SOD1 and Thy1-mitoDsRed (J). Asterisks mark nucleus. MitoDsRed reveals directly the mitochondrial swelling (J, hatched arrows) and fragmentation (J, open arrow) in ALS mouse motor neurons, and magnitude of mitochondrial swelling (K). Scale bars = 2 μm (I) and 2.5 μm (J). **K**. Mitochondrial diameters in motor neurons of Thy1-mitoDsRed2 tg mice (control) and SOD1-G93A:Thy1-mitoDsRed2 tg mice. Values are mean ± SD. Mitochondrial diameters in motor neurons were increased 5-fold (*, p < 0.01, n = 6 mice/group).

To demonstrate the utility of these novel tg mice, they were used in studies of motor neurons. One application was for cell culture and other uses were in vivo. For motor neuron cell culture studies, mitochondrial tracking with Thy1-DsRed expression was combined with whole-cell eGFP labeling. We used tg mice expressing eGFP driven by the Hb9 promoter [17] to identify motor neurons. In spinal cord sections of E12-14 embryos of Hb9-eGFP mice, numerous cells throughout ventral spinal cord express eGFP (Figure 6A). The identification of large-size (> 28 μm) and medium-size (15-28 μm) Hb9-eGFP cells as motor neurons in dissociated spinal cord culture has been confirmed [26,27]. Here, flow cytometry and fluorescence-activated cell sorting (FACS) was used to sort eGFP-expressing motor neurons (Figure 6B, C) for subsequent culture (Figure 6D) and transfection with Thy1-mitoDsRed expression construct to identify mitochondria specifically in living motor neurons (Figure 6F). Another experimental approach was to cross Thy1-mitoDsRed tg mice and Hb9-eGPF tg mice to generate fluorescent protein double tg mouse embryos to directly visualize mitochondria in cultured neurons unequivocally identifiable as motor neurons (Figure 6G, H).

We also crossed tg mice expressing mitoDsRed in neurons and tg mice expressing a human mutant gene that causes motor neuron disease [28-30]. We thus generated double tg with Thy1-mitoDsRed and human mutant SOD1. Motor neuron mitochondria are known to undergo mPTP-dependent pathological swelling and fragmentation in these G93A-SOD1 mice [29,30], and this mitochondrial pathobiology was observed and assessed directly in spinal cord sections of Thy1-mitoDsRed:G93A-SOD1 mice (Figure 6I-K). DsRed-tagged mitochondria in motor neurons of G93A-SOD1 mice showed evidence for both swelling and fragmentation (Figure 6J). Some aberrant motor neuron mitochondria exceeded 3 μm in diameter in these mutant mice (Figure 6K).

**Figure 7:**
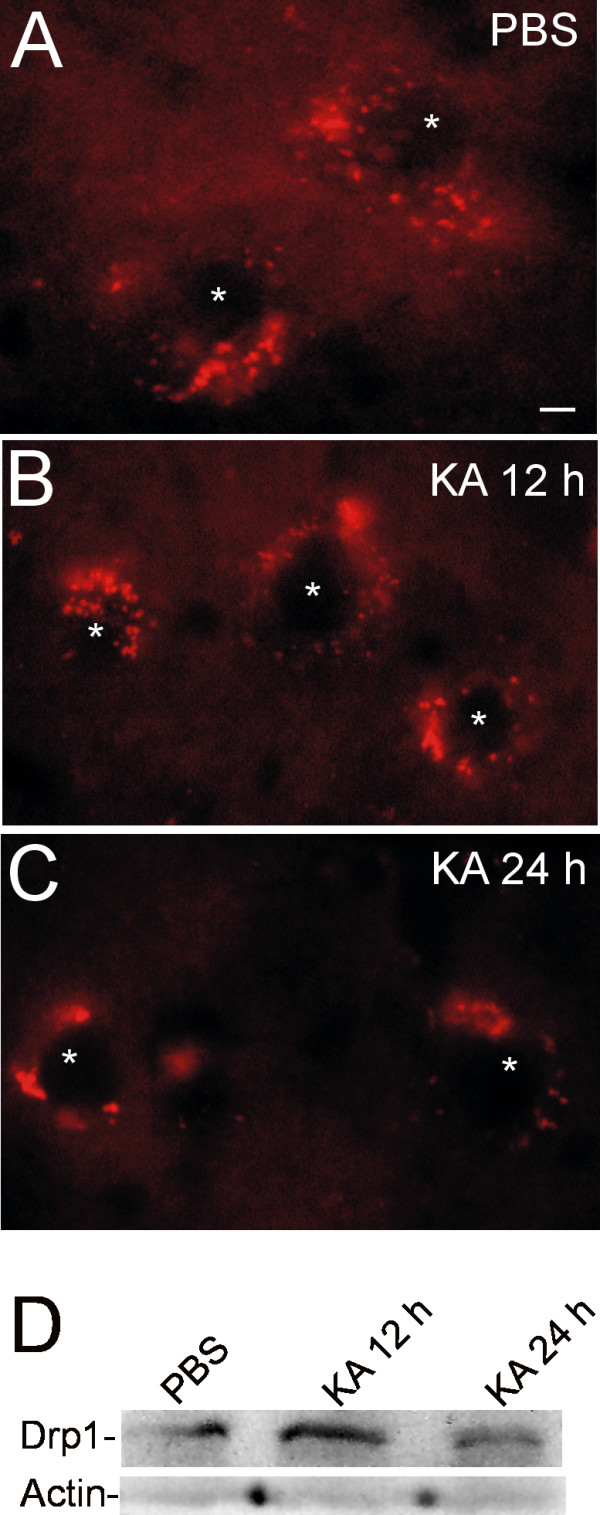
**Application of Thy1-mitoDsRed tg mice in studies of kainic acid (KA) excitotoxicity-induced mitochondrial fission in spinal cord motor neurons**. **A**. Mice with phosphate-buffered saline (PBS) injections placed stereotaxically in lumbar spinal cord had motor neurons with perikaryal (asterisk identifies the nucleus of individual neurons in all images in A-C) DsRed-tagged mitochondria having normal mostly uniform appearances and distributions. **B**. Mice with KA injections in lumbar spinal cord and 12-hour recovery had motor neurons with perikaryal DsRed-tagged mitochondria appearing heterogeneous in size with an apparent increase in small round mitochondria. **C**. Mice with KA injections in lumbar spinal cord and 24-hour recovery had motor neurons with perikaryal DsRed-tagged mitochondria appearing as swollen, clumped or aggregated. Scale bar (in A) = 5 μm (same for B and C). Images are representative of four different tg mice per group. **D**. Western blot for Drp1 in lumbar spinal cord extracts of Thy1-mitoDsRed tg mice after intraspinal injection of PBS or KA and survival for 12 or 24 hours. Actin is shown for protein loading.

To further demonstrate the utility of Thy1-mitoDsRed tg mice, and to reveal novel aspects of mitochondrial biology in stressed neurons, we used an *in vivo *excitotoxicity paradigm (Figure 7). We made stereotaxic microinjections of kainic acid (KA), a potent non-NMDA glutamate agonist [16], into the lumbar spinal cord ventral horn of Thy1-mitoDsRed tg mice and tracked changes in mitochondrial morphology in putative motor neurons in spinal cord sections at different times post-injury. Motor neurons in spinal cords injected with vehicle showed a perikaryal distribution of relatively uniform round, ellipsoid, and vermiform mitochondria (Figure 7A). In contrast, motor neurons undergoing excitotoxic injury had numerous perinuclear mitochondria appearing to undergo fragmentation or fission within 6-12 hours (Figure 7B). At about 12 hours later, perinuclear mitochondria in motor neurons appeared swollen, clumped, or aggregated (Figure 7C), findings similar to those seen in mutant SOD1 tg mice (Figure 6J, K) [30]. To help interpret the morphological findings, western blotting was done for dynamin-related protein 1 (Drp1), a mitochondrial fission protein [31]. The temporal pattern seen for Drp1 in lumbar spinal cord extracts was an upregulation of Drp1 levels at 12 h postlesion, followed by a loss of Drp1 immunoreactivity at 24 postlesion (Figure 7D). The early upregulation of Drp1 is consistent with excitotoxicity-induced mitochondrial fission in motor neurons [16].

## Discussion

We have created new tg mice to study specifically neuronal mitochondria. The construct that we engineered included a *DsRed2 *gene variant with low aggregation properties [32], a Thy1 promoter for neuron-specific expression [33], and a cytochrome *c *oxidase subunit VIII mitochondrial-specific targeting sequence [34]. These tg mice have mitochondria labeled with DsRed in neurons throughout the CNS.

Before going to the expense of zygote microinjection for mouse transgenesis, we ensured that the expression construct worked in cell culture experiments. Thy1-mitoDsRed plasmid DNA was transfected into primary neurons and immortalized neurons and they showed robust DsRed-tagging of mitochondria in live cell culture. Our gold standard comparator for DsRed-labeled mitochondria was the original commercial mito-DsRed expression plasmid used for cloning. The patterns of DsRed labeling were identical with both types of plasmid, except that no astrocyte expression of DsRed was observed with the Thy1-mitoDsRed construct. The observations on mitochondrial distribution and morphology in cultured neural cells gleaned from using our Thy1-mitoDsRed construct are consistent with the literature on neural cell mitochondria [23,24,35]. Further support for the mitochondrial localization of DsRed in neurons in culture was derived from the anticipated pathological changes seen with mitochondria (e.g., swelling and rounding) when neurons received an excitotoxic challenge. Therefore our construct was engineered properly and worked effectively.

The tg mice developed using this construct showed brain- and spinal cord-specific expression of DsRed in mitochondria as determined by western blotting, RT-PCR (except for 1 of 5 mouse lines that showed limited kidney expression), and immunofluoresence for subcellular markers. However, some peripheral organ expression could be due to DsRed in mitochondria of peripheral nerves, but this is seemingly below the limit of protein detection in crude tissue extracts. Subcellular fractionation of CNS tissues confirmed that the DsRed was found the mitochondrial-enriched fractions. The mRNA expression profiles for the *DsRed *gene further confirmed the CNS-specific expression of mitoDsRed. In tissue sections of brain and spinal from tg mice, mitochondrial fluorescence was observed in subsets of neurons. DsRed fluorescent structures in the cell bodies and processes of neurons colocalized with immunodetected mitochondrial markers, and were not found in the nucleus, peroxisomes, or ER. Thus, the creation of tg mice with neuron-specific DsRed-tagged mitochondria was successful. Interestingly, not all mitochondria within an individual neuron were tagged with DsRed, suggesting that the pool of mitochondria within a single neuron is heterogeneous. A within-cell mitochondrial heterogeneity in neurons has been suggested before [36]. We also found that some DsRed-tagged mitochondria in neurons co-labeled with the autophage marker LC3A. Thus, our new tg mice might be useful also for tracking mitophagy in neurons.

Three previous papers have described the generation of tg mice expressing fluorescent proteins in the mitochondria of neurons [37-39]. In an elegant study, conditional bigenic tg mice were created by generating tg mice with a tetracycline responsive element driving mitochondrial targeted enhanced YFP and crossing them to tg mice with calcium/calmodulin-dependent kinase IIα (CaMKIIα)-driven tetracycline transactivator [37]. In histological preparations, these mice showed YFP-tagged mitochondria in neurons of cerebral cortex hippocampus, hippocampus and striatum [37]. Because of the use of the CaMKIIα promoter, only subsets of neurons in forebrain displayed mitochondrial fluorescent protein, unlike our mice which have DsRed-tagged mitochondria in neurons throughout the CNS. This previous study also made the important finding that mitochondria isolated from these mice had normal bioenergetics [37]. Another group created a tg mouse also using the Thy1 promoter, but the mitochondrial-targeted fluorescent protein was cyan fluorescent protein [38] instead of YFP or DsRed. The different tg mouse lines displayed marvelous blue fluorescent mitochondria in distributions throughout the CNS, much like our Thy1-mitoDsRed mice, with the patterns in hippocampus and spinal cord being very similar. More recently, tg mice expressing mitochondrial-targeted eGFP were generated using a CAG promoter driving expression throughout the body, rather than being CNS-specific [39]. Interestingly, these mice [38,39], as in ours, show intense fluorescent labeling in the neuropil. We predict the DsRed fluorescence in the neuropil corresponds to mitochondria in distal dendrites, spines, and presynaptic axon terminals, which needs to be addressed using immunogold electron microscopy using antibody to DsRed. However, before such a task in undertaken, we need to better characterize the DsRed antibody used here for suitability in immunohistochemical applications, or use different reliable antibodies to DsRed. In any event, Lichtman and colleagues [38] addressed the issue of the non-somal fluorescent protein-tagged mitochondria by using live cell time-lapse imaging of peripheral nerve axons and demonstrating magnificently the trafficking of mitochondria in axons.

We demonstrated how neurons with mitochondrial targeted DsRed tg can be useful to the study of motor neurons, and novel aspects of their mitochondrial biology, in four different novel paradigms. In one experiment, we used FACS to isolate spinal cord motor neurons from Hb9-eGFP tg mouse embryos. Viable eGFP-positive motor neurons were cultured and then transfected with Thy1-mitoDsRed construct to visualize mitochondria specifically in a relatively pure population of motor neurons in cell culture. This idea was pursued in a second experiment by crossing Hb9-eGFP tg mice and Thy1-mitoDsRed tg mice. Primary embryonic spinal cord cultures were prepared to visualize eGFP/mitoDsRed dual-labeled motor neurons. In a third experimental design, Thy1-mitoDsRed tg were crossed to tg mice expressing human mutant SOD1. Tg mice expressing human mutated *SOD1 *genes develop fatal motor neuron disease [28] with severe mitochondrial pathology as detected with biochemical methods, antibodies to mitochondrial proteins, and electron microscopy [29,30,40]. Double tg mice expressing mutant SOD1 and mito-DsRed reveal directly the mitochondriopathy in motor neurons without the aid of immunohistochemistry or electron microscopy. Motor neurons in these ALS mice showed evidence for severe mitochondrial swelling and fragmentation or possible fission. Similar experiments are planned for crossing Thy1-mitoDsRed tg mice to mouse models of Parkinson's disease [41] and Alzheimer's disease [42] to further pursue mitochondrial-based mechanisms of disease.

Thy1-mitoDsRed tg mice were used in a fourth experimental application involving spinal cord excitotoxic injury. We have reported previously that excitotoxic activation of glutamate receptors on spinal cord motor neurons *in vivo *induces rapid fragmentation or fission of mitochondria mediated by activation of the mPTP [16]. Our experiments here confirm and extend this finding by visualizing these mitochondrial morphology changes directly and showing that the abnormality is associated with an upregulation of Drp1. This latter result suggests that excitotoxicity *in vivo *acutely induces mitochondrial fission in motor neurons. However, as the injury evolves mitochondria may undergo fusion to form large abnormal aggregates or clusters of mitochondria within the perikaryon of motor neurons.

## Conclusions

This is the first demonstration of the generation of a tg mouse with DsRed protein expressed selectively in mitochondria of neurons throughout the CNS. We describe the development and biochemical and histological characterization of these mice. This new tg mouse has broad application for studies of neuronal mitochondrial biology and the involvement of neuronal mitochondria in the cellular and molecular mechanisms of neurodegeneration in mouse and cell models of neurological disease. The applications of these new tg mice provide novel insights into the pathobiology of mitochondria in neurons, such as their swelling and fission, and are relevant to ALS.

## Methods

### Design of Thy1-mitoDsRed gene construct

We used a Thy-1 promoter (generously provided by Dr. Pico Caroni) to drive DsRed2 expression in a neuron-specific pattern [33]. This promoter has been used to create several lines of tg mice with neuron-specific expression of exogenous proteins [43,44]. The Thy1 regulatory element for expression is composed of a 4.1 kb fragment obtained from the 5' portion of the *Thy1 *gene extending from the promoter to the intron following exon 4, but exon 3 and its flanking introns are absent [33]. These sequence deletions are responsible for neuron-specific expression and the absence of expression in non-neural cells. DsRed2 is a genetic variant of DsRed modified with six point mutations (Clontech Laboratories, Mountain View, CA). These mutations improve the solubility of DsRed2 by reducing its tendency to form aggregates [32]. DsRed2 retains the benefits typical of red fluorescent proteins, such as low or no autofluorescence and high signal-to-noise ratio, and is optimized for compatible expression in mammalian cells as a reporter gene [32]. The vector used was pDsRed2-mito (Clontech). This vector encodes the full-length *Dicosoma sp*. red fluorescent protein and the mitochondrial targeting sequence from subunit VIII of human cytochrome *c *oxidase [34]. The mitochondrial targeting sequence was ligated in frame to the 5' end of the *DsRed2 *gene to direct the protein expression of DsRed2 only in the mitochondria of cells.

### Neuronal cell culture and transfection

The NSC34 cell line was obtained from Cellutions Biosystems Inc (Ontario, Canada). NSC34 cells are a mouse spinal cord cell line created by fusing embryonic primary spinal cord cells with neuroblastoma cells [45]. Under appropriate culture conditions NSC34 cells can differentiate into motor neuron-like cells [46]. NSC34 cells were maintained and subcultured every 3 days in DMEM with 10% fetal bovine serum. NSC34 cell differentiation culture medium consisted of high-glucose DMEM supplemented with 10% heat-inactivated fetal bovine serum (Invitrogen, Carlsbad, CA), 2 mM L-glutamine, 0.1 mM nonessential amino acids, and antibiotics at 37°C in 95% air-5% CO_2_. NSC34 cells used for transfection experiments were cultured between 5-10 days *in vitro *(DIV).

All protocols (MO10321) using animals were approved by the Animal Use and Care Committee of the Johns Hopkins University School of Medicine. Primary embryonic motor neuron and cortical neuron cultures were prepared from timed-pregnant tg mice (B6.Cg-Tg (Hlxb9-gfp)1Tmj/j) expressing eGFP driven by the mouse *Hlxb9 *(Hb9) promoter [17] obtained from The Jackson Laboratory (Bar Harbor, Maine) and from Thy1-mitoDsRed2 tg mice (see below for generation). Hb9 is a homeodomain transcription factor that is expressed by motor neurons and functions during development to consolidate motor neuron identity [47,48]. To obtain embryos for dissociated spinal cord neuron and cortical neuron culture, gestational day 12-14 (E12-14), female mice with potential tg Hb9-eGFP or Thy1-mitoDsRed2 embryos were anesthetized with isoflurane and all embryos were harvested by caesarian section. Hb9-eGFP expression in embryos was confirmed under a fluorescence microscope. Primary motor neuron cultures were obtained from total spinal cords of male and female Hb9-eGFP^+ ^embryos. Primary cortical neuron cultures were obtained from total cerebral cortices of male and female embryos irrespective of eGFP or DsRed genotype. The whole spinal cords and cerebral cortices were dissected and incubated for 30 min in 0. 025% trypsin and then dissociated by gentle trituration. Trypsin-digested spinal cords were washed in HBSS and sorted by fluorescence-activated cell sorting (FACS) using a FACSVantage SE flow cytometer equipped with an Argon laser for excitation at 488 nm. The resulting pure motor neurons or mixed cortical cells were plated on poly-D-lysine/laminin-coated glass coverslips (12 mm in diameter; BD Transduction Laboratories, Franklin Lakes, NJ) or chamber slides and maintained in Dulbecco's modified Eagle's medium (DMEM) supplemented with 5% fetal bovine serum and 5% horse serum (Invitrogen). At 4-6 h after plating, the medium was replaced with Neurobasal supplemented with B-27 (Invitrogen) or with DMEM supplemented with 10% horse serum. On the second day after plating, uridine and 5-fluoro-2-deoxyuridine were added to the culture medium to inhibit the proliferation of contaminating non-neuronal cells. The motor neurons and cortical neurons were used for transfection or excitotoxicity experiments on DIV12-16.

Transient transfections of NSC34 cells and primary motor neuron and cortical neurons with Thy1-mitoDsRed2 expression plasmids were performed using Lipofectamine 2000 (Invitrogen). As a positive control NSC34 cells and primary neurons were transfected with the original mitoDsRed2 plasmid with a CMV promoter (Clontech) which was used to engineer our construct. Live cells were imaged using epifluorescence microscopy 48 h later and were then fixed for 20 min in 4% paraformaldehyde/4% sucrose.

Thy1-mitoDsRed cortical neurons grown in DMEM and horse serum were subjected to excitotoxic stress by adding the highly-specific *N*-methyl-*D*-aspartate glutamate receptor agonist quinolinic acid to the media at a final concentration of 100 μM. Live cells were imaged using epifluorescence microscopy 4-24 h later.

### Generation of Thy1-mitoDsRed2 tg mice

To create tg mice, pUC18 plasmid was restriction enzyme digested, resulting in a 7.1 Kb fragment. The fragments were given to the Johns Hopkins Transgenic Core Facility for zygote-injection-based transgenesis using B6SJLF1 mouse embryos. The institutional Animal Care and Use Committee approved the animal protocols. Potential founders were screened by PCR analysis of tail genomic DNA using 3 different primer pairs. Mouse tails were digested using DirectPCR Lysis Reagent (Viagen Biotech, Los Angeles, CA) and DNA was extracted from the lysate by precipitation with isopropanol. The presence of the *mito-DsRed *transgene was identified with the following sets of primers:

5'-GACCCACAAGGCCCTGAAG-3' and 5'-TGCTCCACGATGGTGTAGTCC-3' (product 719 bp); 5'-ATGGCCTCCTCCGAGAACGTCATC-3' and 5'-GGTACCGTCGACTGCAGAATTCGA-3' (product 715 bp); 5'- GTTCCAGTACGGCTCCAAGGTGTA-3' and 5'-ATGGTGTTAGTCCTCGTTGTGGGAG-3' (product 438 bp); or 5'-CCCCGTAATGCAGAAGAAGA-3' and 5'-GGTGATGTCCAGTTGGAGT-3' (product 208 bp). All PCR products contained a coding region of *DsRed2 *gene. For an internal control the *interleukin-2 *gene was amplified with the following primers: 5'-CTAGGCCACAGAATTGAAAGATCT-3' and 5'-GTAGGTGGAAATTCTAGCATCATCC-3' (product 324 bp).

PCR was performed using a Techne thermocycler with a 2 min preheating at 95°C, followed by 35 cycles of denaturation at 95°C, 1 min, annealing at 55°C, 1 min and extension at 72°C, 1 min. Amplification was completed with an additional 7 min extension at 72°C. PCR products were resolve by agarose gel electrophoresis and stained with ethidium bromide.

### Tissue harvesting for protein and mRNA expression

Thy1-mitoDsRed2 tg and non-tg mice at 2-4 months of age (n = 4-6/genotype) and 10-12 months of age (n = 4-6/genotype) were killed by CO_2 _inhalation, and the following tissues and organs were quickly harvested and frozen on dry ice: brain, spinal cord, hindleg skeletal muscle, liver, kidney, and heart. Before freezing some brains were also microdissected in ice-cold media into the following regions: olfactory bulb, cerebral cortex, hippocampus, striatum, diencephalon, brainstem, and cerebellum.

### Immunoblotting

Western blot analysis was performed to examine expression of DsRed in CNS and systemic organs. Tissue samples were pulverized and homogenized with a Brinkmann polytron in ice-cold 20 mM Tris HCl (pH 7.4) containing 10% (wt/vol) sucrose, 200 mM mannitol, complete protease inhibitor cocktail (Roche, Indianapolis, IN), 0.1 mM phenylmethylsulfonyl fluoride, 10 mM benzamidine, 1 mM EDTA, and 5 mM EGTA. Crude homogenates were sonicated for 15 sec and then centrifuged at 1, 000 g_av _for 10 min (4°C). The supernatant was centrifuged at 54, 000 g_av _for 20 minutes (4°C) to yield soluble (S2) and mitochondria-enriched pellet (P2) fractions. This subcellular fractionation protocol has been verified [49]. The pellet fraction was washed (twice) by trituration in homogenization buffer followed by centrifugation and then finally resuspended in homogenization buffer (without sucrose) supplemented with 20% (wt/vol) glycerol. Protein concentrations were measured by a Bio-Rad protein assay with bovine serum albumin as a standard.

Tissue protein extracts from Thy1-mitoDsRed2 tg mice and non-tg mice were subjected to sodium dodecyl sulfate polyacrylamide gel electrophoresis (SDS-PAGE) and transferred to nitrocellulose membrane by electroelution as described [49]. Recombinant DsRed protein (Clontech) was loaded as a positive control. The reliability of sample loading and electroblotting in each experiment was evaluated by staining nitrocellulose membranes with Ponceau S before immunoblotting. Ponceau S stained membranes were scanned for documentation of protein loading and even transfer among lanes. If transfer was not uniform, blots were discarded and gels were run again. Blots were blocked with 2.5% nonfat dry milk with 0.1% Tween 20 in 50 mM Tris-buffered saline (pH 7.4), then incubated overnight at 4°C with antibodies to DsRed that were either rabbit polyclonal antibodies (BioVision Research Products, Mountain View, CA) or mouse monoclonal antibody (Clontech). The antibodies were used at concentrations for visualizing protein immunoreactivity within the linear range. As a protein loading control, some blots were re-probed with monoclonal antibody to cofilin (Sigma-Aldrich, St Louis, MO). After the primary antibody incubation, blots were washed and incubated with horseradish peroxidase (HRP)-conjugated secondary antibody (0.2 μg/ml), developed with enhanced chemiluminescence (Pierce), and exposed to x-ray film.

### Reverse transcription-polymerase chain reaction (RT-PCR)

To corroborate findings based on DsRed antibody approaches, RT-PCR was used to analyze mRNA expression for DsRed in Thy1-mitoDsRed tg mice. Total RNA was extracted using TRIzol (Invitrogen) from mouse CNS and peripheral organs. cDNA synthesis was accomplished using SuperScript One-Step RT-PCR with Platinum *Taq *(Invitrogen) followed by PCR. The oligonucleotide primer pair used to amplify a DsRed cDNA 435 bp product was: sense, 5'-CTGTCCCCCCAGTTCCAGTAC-3; antisense, 5'-cgttgtgggaggtgatgtccagct-3' [50]. For a total RNA control for each sample, oligonucleotide primers for mouse voltage-dependent anion channel-1 (VDAC1) were 5'-GCTAAGGATGACTCGGCTTTAAGG-3' and 5'-AGGTTAAGTGATGGGCTAGGATGG-3' which give a 335 bp amplification product [51]. The PCR products were separated on a 1.0% agarose gel stained with ethidium bromide and imaged using a BioRad molecular imaging VersaDoc system.

### Histology and immunohistochemistry

Thy1-mitoDsRed tg mice and age-match non-tg littermate control mice at 1 month, 6 months and 12 months of age(n = 6-10/genotype/time) were deeply anesthetized and perfusion-fixed using 4% paraformaldehyde. After perfusion-fixation, the brain and spinal cord were removed from each mouse, and the tissues were cryoprotected (20% glycerol). The brains and lumbar spinal cords were frozen-sectioned (40 μm) in the transverse plane using a sliding microtome. Serial section arrays were stored individually in 96-well plates. Every 10^th ^section of brain and spinal cord was mounted on glass slides, coverslipped with antifade mounting medium, and viewed using epifluorescence or confocal microscopy.

To characterize the intracellular localization of DsRed in tg mice known markers for organelles were used for dual labeling. The mitochondrial outer membrane voltage-dependent anion channel (VDAC) was detected with a mouse monoclonal antibody (MitoScience, Eugene, OR) diluted at 1:500. The mitochondrial matrix protein manganese superoxide dismutase (MnSOD, SOD2) was detected with two different rabbit polyclonal antibodies (SOD-110 and SOD-111, Stressgen, Victoria, British Columbia, Canada) diluted at 1:500. The peroxisome and nuclear compartments were visualized with a sheep polyclonal antibody to catalase (Biodesign international, Saco, ME) diluted at 1:500. The ER compartment was visualized with a rabbit polyclonal antibody to cytochrome P450 reductase (Stressgen) diluted at 1:500. Immunoreactivities were visualized with an AlexaFluor-488 conjugated secondary antibody and counterstained with Hoechst-33342 dye for nuclear identification.

To determine if DsRed2 expression caused neurotoxicity in tg mice, brain and spinal cord sections were also examined for pathology. Sections were examined using Nissl staining with cresyl violet, silver staining using the FD NeuroSilver kit (FD Neurotechnologies Inc, Baltimore, MD), and immunofluorescent staining for cleaved caspase-3 detected with a rabbit polyclonal antibody (1:1000) to the active subunits (Cell Signaling, Beverly, MA) and AlexaFluor-488 conjugated secondary antibody.

### Double tg mice

Two different lines of tg mice were crossed to Thy1-mitoDsRed tg mice. To study mitochondria in specifically motor neurons, Thy1-mitoDsRed tg mice were crossed to Hb9-eGFP tg mice. To study mitochondria in a mouse model of ALS, Thy1-mitoDsRed tg mice were crossed to human G93A-mutant SOD1 tg mice [29,30]. F1 offspring positive for the DsRed and eGFP transgenes or DsRed and human SOD1 transgenes were identified by PCR. Histology was done as described above. Mitochondrial diameter measurements in motor neurons were done as described [29]. Small-particle, mitochondrial fragment-like structures were not measured due to resolution limits.

### Excitotoxic lesions in mouse spinal cord

Adult Thy1-mitoDsRed tg mice were anesthetized deeply with isoflurane (4%) in O_2 _in an induction chamber, and the lower back was shaved of fur and cleaned with betadine and 70% isopropyl alcohol. Mice were mounted in a stereotaxic apparatus (Stoelting, Wood Dale, IL) with a customized vertebral column stabilizer. Surgical anesthesia was maintained with isoflurane (2%), nitrous oxide (66%), and O_2 _(32%) delivered by a nosepiece mask. Body temperature was maintained with external warming. A midsagittal incision (4 mm) was made over the lumbar back, and the dorsal part of the L1-L3 vertebral column was carefully freed of attached fascia and skeletal muscle. After a laminectomy at L2, performed slowly and carefully without traumatizing the spinal cord and causing any edema, a stereotaxic injection of the non-NMDA ion channel glutamate receptor agonist kainic acid (KA, 400 μmol, 1 μl volume) was made unilaterally directly into the parenchyma of lumbar spinal cord using a mounted 10-μl Hamilton syringe and a sharp, stainless steel needle (26 gauge) with a 45° beveled angle. KA (Sigma) was dissolved in 100 mM phosphate-buffered saline (PBS, pH 7.4) and was stored in the dark at -20°C until used. The dose of KA was established previously [16]. Mice injected unilaterally with 100 mM PBS (1 μl) were controls. Injections were performed manually. To prevent leakage from the injection site, neurotoxin and buffer were injected over 1 min, and the needle was left in place for 3 min before it was withdrawn slowly. The laminectomy was sealed with softened W-31 bone wax (Ethicon, Somerville, NJ), and the wound was closed with a skin clip. Postoperative care was as described [16].

At 6, 12, and 24 hours after the excitotoxic injection, mice (n = 4/time) were deeply anesthetized with an overdose of sodium pentobarbital and either perfused intra-cardially with ice-cold PBS (100 mM, pH 7.4) followed by ice-cold 4% paraformaldehyde in PBS or were killed for harvesting of fresh unfixed spinal cord. After perfusion-fixation, spinal cords remained *in situ *for 2 hours before they were removed by dorsal laminectomy from the vertebral column. The lumbar enlargements were cryoprotected in 20% glycerol-PBS, and frozen under pulverized dry ice. Transverse cryosections (40 μm) were cut using a sliding microtome, mounted on glass slides, and viewed using epifluorescence microscopy. Fresh lumbar spinal cord samples were homogenized and used for western blotting as described above. Blots were probed for Drp1 using a rabbit polyclonal antibody (ProteinTech Group, Chicago, IL) diluted at 1:800, then for actin as a loading control.

### Photography and figure construction

The original images used for figure construction were generated using digital photography. Digital images were captured as TiFF files using a SPOT digital camera and SPOT Advanced software (Diagnostic Instruments) or a Nikon digital camera (DXM1200) and ACT-1 software. Images were altered slightly for brightness and contrast using ArcSoft PhotoStudio 2000 or Adobe Photoshop software without changing the content and actual result. Figure composition was done using CorelDraw 9 software with final figures being converted to TiFF files. Files of composite figures were adjusted for brightness and contrast in Adobe Photoshop.

## Competing interests

The authors declare that they have no competing interests.

## Authors' contributions

YW and LJM conceived and designed the experiments. YW made the construct. LJM wrote the manuscript. YP and AP contributed to the biochemical and histological characterization of the transgenic mice. All authors approved the final manuscript.
